# Leitplanken statt Sperrbalken

**DOI:** 10.1007/s00103-022-03646-4

**Published:** 2023-02-01

**Authors:** Stefanie Weber, Joseph Kuhn

**Affiliations:** 1 Abteilung „Kodiersysteme und Register“, Bundesinstitut für Arzneimittel und Medizinprodukte, Kurt-Georg-Kiesinger-Allee 3, 53175 Bonn, Deutschland; 2 Bayerisches Landesamt für Gesundheit und Lebensmittelsicherheit, Veterinärstr. 2, 85764 Oberschleißheim, Deutschland

Digitalisierung ist eines der Zauberwörter für die Entwicklung des Gesundheitswesens im 21. Jahrhundert. Dabei ist allen bewusst, dass vor der Erfüllung der Hoffnungen ein steiniger Weg kommt. Technische und rechtliche Hürden spielen dabei eine wichtige Rolle, aber auch Interessenskonflikte unter den Beteiligten und – was oft zu wenig beachtet wird – Unklarheiten über die strategischen Ziele der Digitalisierung im Gesundheitswesen. Es geht nicht nur um eine technologische Innovation, sondern auch darum, welche Fortschritte in der Versorgungsqualität durch Digitalisierung erreicht werden sollen und können. Daran orientiert stellt dann die Gestaltung der technischen, rechtlichen und ethischen Rahmenbedingungen einen wichtigen Faktor der Digitalisierung im Gesundheitswesen dar. Diese setzen die Eckpfeiler für Forschungsvorhaben, Implementierungsinitiativen und Modellprojekte.

Die technischen, rechtlichen und ethischen Rahmenbedingungen der Digitalisierung lassen sich nicht systematisch über alle Handlungsfelder hinweg abhandeln. Aber anhand ausgewählter Vorhaben lassen sich die Relevanz dieser Aspekte und ihre Verzahnung untereinander aufzeigen. Die Corona-Pandemie hat beispielsweise die Notwendigkeit eines schnellen Zugangs zu Gesundheitsdaten für politische Entscheidungen gezeigt und der Digitalisierung einen kräftigen Schub gegeben. Dies betrifft die Gesundheitsforschung, die Versorgung und die Gesundheitsverwaltung gleichermaßen. Im öffentlichen Gesundheitsdienst (ÖGD) werden über den „Pakt für den ÖGD“ 800 Mio. € für die Digitalisierung bereitgestellt. Die Summe mag man auch als Maß für den Nachholbedarf sehen.

In der Gesundheitsversorgung beginnen sich neue digitale Behandlungspfade, wie Videosprechstunden, zunehmend zu etablieren. Sie sind auf nutzerfreundliche Technik und rechtssichere Rahmenbedingungen angewiesen. Technische und wissenschaftliche Fortschritte wie die künstliche Intelligenz eröffnen neue Forschungs- und Steuerungsoptionen. Damit verbunden sind wiederum grundlegende ethische wie rechtliche Herausforderungen: Wenn sich Entscheidungen auf Prozesse der künstlichen Intelligenz stützen, wer steht dann eigentlich in der Verantwortung? Und führt der Datenreichtum im Gesundheitswesen, der über die Digitalisierung erschlossen werden kann, immer zu einem Mehr an Erkenntnisgewinn oder mitunter auch zu mehr Heu um die Nadel im Heuhaufen? Was folgt daraus für das Zusammenspiel von Studiendesigns, aber auch für die Forschungsethik?

Die beschleunigte Entwicklung der Digitalisierung soll in diesem Themenheft anhand von diskursprägenden Beispielen mit einem Fokus auf technische, rechtliche und ethische Rahmenbedingungen beleuchtet werden. Das Heft ist Teil einer Reihe des *Bundesgesundheitsblatts* zur Digitalisierung im Gesundheitswesen und hat daher nicht den Anspruch, die ganze Breite der diskussionsbedürftigen Themen rund um die Digitalisierung abzubilden. Wichtige Themen, z. B. was die bessere Verfügbarkeit von Daten als „Treibstoff“ der Digitalisierung angeht, oder die Notwendigkeit, Aus- und Weiterbildungsinhalte im Gesundheitswesen an die Digitalisierung anzupassen, sollen in anderen Ausgaben angesprochen werden.

Als Einstieg in das Themenheft schildern *Stachwitz und Debatin* in einem Übersichtsartikel den Status quo der Telematikinfrastruktur. Dabei beleuchten sie auch den Sinn, die Hürden und die Kompromisse im Verlauf der Entwicklung.

Anhand von 3 Beispielen wird dann der Fokus auf die technischen Rahmenbedingungen gelegt. Am Beispiel der Forschungsinitiativen zu COVID-19 des Netzwerks Universitätsmedizin werden von *Heyder et al.* verschiedene Teilaspekte der Datengewinnung und -auswertung mit Fokus auf die technisch-organisatorischen Aspekte beschrieben und bewertet. Durch unterschiedliche Ansätze konnten gut geeignete Modelle identifiziert werden. Im Artikel von *Flöter et al.* wird dann ganz konkret am Beispiel des bayerischen Meldeportals für Corona-Reihenuntersuchungen eine Digitalisierungsaktivität beschrieben, die unter großem Zeitdruck entstanden ist. Der Artikel analysiert die Stärken und Schwächen des Vorgehens und die manchmal abweichende Erwartungshaltung gegenüber der technischen Realisierbarkeit. Im dritten Artikel des Blocks wird von *Eymann et al.* ein Modell zur Bewertung der digitalen Reife des ÖGD vorgestellt. Wichtig für den Fortschritt der Digitalisierung ist nicht nur die Einführung von Neuerungen, sondern auch die stetige Bewertung, Reflektion und ggf. Anpassung der technischen Lösungen.

Im Block zu den rechtlichen Rahmenbedingungen steht, wie zu erwarten, das Thema Datenschutz im Fokus. Mit einer Bewertung der EU-Datenschutz-Grundverordnung und des darin enthaltenen risikobasierten Ansatzes zum Datenschutz gibt *Molnár-Gábor* einen Denkanstoß aus juristischer Sicht für die praktische Umsetzung der Digitalisierung. Auch im Hinblick auf die laufende Gesetzgebung zum Europäischen Gesundheitsdatenraum ist dies ein Thema, das in den nächsten Jahren sicher den rechtlichen Rahmen der Digitalisierung maßgeblich beeinflussen wird. Im zweiten Artikel wird dann der Blick auf die praktischen Fragen zu rechtlichen Rahmenbedingungen anhand der Videosprechstunde gelenkt. *Steubl und Baumeister* nehmen dabei nicht nur die datenschutzrechtlichen Aspekte, sondern auch ganz praktische Fragen, wie z. B. das Abrechnungsrecht, in den Blick, das noch nicht immer den Anforderungen der geänderten Rahmenbedingungen durch die Digitalisierung gerecht wird und manchmal gar als eine Hürde empfunden wird.

Im letzten Block wird ein Blick auf die ethischen Rahmenbedingungen geworfen. Der Beitrag von *Schickhardt et al.* beschäftigt sich mit der digitalen Infrastruktur bei großen Datenprojekten. Inwiefern ist diese ebenfalls ein Akteur im Gesamtgeschehen und welche Verantwortung kommt ihr dabei zu? Die ethische Betrachtung dieser Fragestellung sollte dabei nicht zuletzt dem Vertrauen der Betroffenen in die sicherere und zweckgebundene Verwendung ihrer Daten Rechnung tragen. Im Beitrag von *Jansky et al.* wird der Aspekt der sozialen Gesundheitsgerechtigkeit am Beispiel von mobilen Gesundheitstechnologien (M-Health-Anwendungen) beleuchtet. Durch die Förderung, aber auch die Forderung der Eigenverantwortlichkeit kann es zur Benachteiligung von vulnerablen Gruppen kommen – eine ethisch nicht unbedenkliche Entwicklung im Rahmen der Digitalisierung. Und schließlich schauen *Küster und Schultz* im letzten Artikel dieses Themenheftes auf die Frage der Symbiose oder des Spagats zwischen Ethik und künstlicher Intelligenz (KI). Gerade im Gesundheitswesen kann künstliche Intelligenz große medizinische Fortschritte bedingen, löst aber auch große Ängste aus. Am Beispiel der Entwicklung eines KI-basierten Empfehlungssystems zur tertiären Prävention von Demenz plädiert der Beitrag für einen langfristig ethisch basierten Ansatz.

Wir hoffen, mit den verschiedenen Sichtweisen auf die Rahmenbedingungen der Digitalisierung Ihr Interesse geweckt zu haben und Sie zum Nachdenken anregen zu können. Letztendlich kann die Digitalisierung nur ein Mittel zum Zweck der besseren Versorgung für uns alle darstellen. Die Rahmenbedingungen sind hierbei oft noch hinderlich und werden manchmal als Sperrbalken empfunden, doch können sie durch beständige Reflexion und Diskussion so angepasst und verbessert werden, dass sie hilfreiche Leitplanken darstellen und neuen Digitalisierungsschritten den Weg bereiten.

